# A hybrid learning framework for fine-grained interpretation of brain spatiotemporal patterns during naturalistic functional magnetic resonance imaging

**DOI:** 10.3389/fnhum.2022.944543

**Published:** 2022-09-30

**Authors:** Sigang Yu, Enze Shi, Ruoyang Wang, Shijie Zhao, Tianming Liu, Xi Jiang, Shu Zhang

**Affiliations:** ^1^Center for Brain and Brain-Inspired Computing Research, Department of Computer Science, Northwestern Polytechnical University, Xi’an, China; ^2^School of Automation, Northwestern Polytechnical University, Xi’an, China; ^3^Cortical Architecture Imaging and Discovery Lab, Department of Computer Science and Bioimaging Research Center, The University of Georgia, Athens, GA, United States; ^4^The Clinical Hospital of Chengdu Brain Science Institute, MOE Key Lab for Neuroinformation, School of Life Science and Technology, University of Electronic Science and Technology of China, Chengdu, China

**Keywords:** naturalistic stimuli, spatiotemporal, fMRI, convolutional neural network, Predictive Model

## Abstract

Naturalistic stimuli, including movie, music, and speech, have been increasingly applied in the research of neuroimaging. Relative to a resting-state or single-task state, naturalistic stimuli can evoke more intense brain activities and have been proved to possess higher test–retest reliability, suggesting greater potential to study adaptive human brain function. In the current research, naturalistic functional magnetic resonance imaging (N-fMRI) has been a powerful tool to record brain states under naturalistic stimuli, and many efforts have been devoted to study the high-level semantic features from spatial or temporal representations *via* N-fMRI. However, integrating both spatial and temporal characteristics of brain activities for better interpreting the patterns under naturalistic stimuli is still underexplored. In this work, a novel hybrid learning framework that comprehensively investigates both the spatial (*via* Predictive Model) and the temporal [*via* convolutional neural network (CNN) model] characteristics of the brain is proposed. Specifically, to focus on certain relevant regions from the whole brain, regions of significance (ROS), which contain common spatial activation characteristics across individuals, are selected *via* the Predictive Model. Further, voxels of significance (VOS), whose signals contain significant temporal characteristics under naturalistic stimuli, are interpreted *via* one-dimensional CNN (1D-CNN) model. In this article, our proposed framework is applied onto the N-fMRI data during naturalistic classical/pop/speech audios stimuli. The promising performance is achieved *via* the Predictive Model to differentiate the different audio categories. Especially for distinguishing the classic and speech audios, the accuracy of classification is up to 92%. Moreover, spatial ROS and VOS are effectively obtained. Besides, temporal characteristics of the high-level semantic features are investigated on the frequency domain *via* convolution kernels of 1D-CNN model, and we effectively bridge the “semantic gap” between high-level semantic features of N-fMRI and low-level acoustic features of naturalistic audios in the frequency domain. Our results provide novel insights on characterizing spatiotemporal patterns of brain activities *via* N-fMRI and effectively explore the high-level semantic features under naturalistic stimuli, which will further benefit the understanding of the brain working mechanism and the advance of naturalistic stimuli clinical application.

## Introduction

Naturalistic stimuli, including movie, music, and speech, are close to real-life experience for human and have been increasingly applied in the research field of neuroimaging (Vanderwal et al., 2019; Wang et al., 2020; Saarimäki, 2021). Relative to a resting-state or single-task state, naturalistic stimuli can evoke more intense brain activities and have been proved to possess higher test–retest reliability, suggesting greater potential to study adaptive human brain function (Martinez-Garcia et al., 2012; Sonkusare et al., 2019; Simony and Chang, 2020). Especially, naturalistic audios, containing rich dynamic auditory stimuli, have been widely adopted as stimulus materials in brain function analysis. To study the functional characteristics of brain activities under naturalistic auditory stimuli, brain imaging, e.g., naturalistic functional magnetic resonance imaging (N-fMRI), has shown great potential to record the brain states and bring more explanations for the brain working mechanism (Saarimäki, 2021).

In recent years, researchers have presented many interesting findings with N-fMRI, including brain function and data reliability. For example, Lahnakoski et al. (2012) explored the detailed relationship between superior temporal sulcus (STS) and social features during watching movie clips with preselected social signals. Wang et al. (2017) proved that the reliability of connectivity and graph theoretical measures of brain networks is significantly improved during naturalistic stimuli over resting-state. Shain et al. (2020) found that human mechanisms generate predictions about upcoming words of a naturalistic sentence mainly by cognitive processes. Although these researches brought a lot of new views, they had a limited contribution to bridge the “semantic gap” (Ozcelik et al., 2022; Raposo et al., 2022). To be specific, “semantic gap” between high-level semantic features and low-level acoustic features is still large, where the former is features contained in N-fMRI with the high-level perception of human, and the latter is features merely extracted from the audios according to dynamics, rhythm, timber, pitch, and tonal (Jiang et al., 2012; Zhao et al., 2014; Lad and Patel, 2021).

In order to interpret the brain conditions and obtain the significant high-level semantic features from fMRI, researchers have made great efforts in terms of spatial and temporal analysis of brain activities. For the spatial analysis, Jiang et al. (2012) developed a computational framework to model the brain imaging space (BIS) high-level features from fMRI and achieved well classification accuracy to differentiate music/speech audios. Zhao et al. (2014) adopted brain network components to decode biologically plausible auditory saliency and effectively decoded the auditory saliency features. Hu et al. (2015) explored the brain regions and functional interactions during semantics categorization based on sparse multinomial logistic regression (SMLR) algorithm. Çelik et al. (2019) proposed a spatially informed voxel-wise modeling (SPIN-VM) technique and achieved well sensitivity in the assessment of fine-grained cortical representations. Zhang et al. (2021) developed a voxel-based state space modeling method and achieved a better understanding of high-dimensional brain activity elicited by complex, open-ended naturalistic tasks. For the temporal analysis, Chen and Hu (2018) applied recurrent neural network (RNN) model based on GRU to capture the sequential information in fMRI data, which can extract GRU patterns and identify subjects. Yan et al. (2019) proposed a multiscale RNN model, using time courses of fMRI independent components directly and achieving promising performance on classification task. Wang et al. (2020) explored functional brain networks underlying auditory saliency *via* a multivariate brain decoding approach. Moreover, much evidence has been proposed that integration of both brain spatial and temporal representations can benefit interpreting the brain state and improve the characterization of the patterns of different brain states. Zhang Y. et al. (2019) proposed a two-stage deep belief network (DBN)–based blind source separation (BSS) method and used it to explore functional brain networks in naturalistic fMRI data. Ren et al. (2021) proposed a volumetric neural architecture search and deep belief network (NAS-DBN) framework to model the N-fMRI volume images, which uncover the hierarchical temporal responses and spatial distributions at multiple scales under naturalistic stimuli. These works extracted both spatial and temporal features from fMRI by various frameworks and achieved satisfying performance, especially for the deep learning–based framework. However, due to the limited size and considerable individual difference of N-fMRI, an efficient and effective framework, which can overcome the issue of the small dataset and retain reasonable classification and prediction performance, is largely needed. Therefore, we aim to study the fine-grained interpretation of brain spatiotemporal patterns on the voxel level, which can benefit to bridge the “semantic gap.”

To design a suitable framework, choosing appropriate learning algorithms is the key issue for differentiating the brain states and identifying meaningful biomarkers. With the rapid development of deep learning and prediction approaches, they have shown the great power of explanation and generalization in human neuroscience (Rosenberg et al., 2018; Wen et al., 2018). Traditionally, studies on fMRI mainly focused on finding correlations between brain and stimulus. The generated models can work well on trained individual data, but they are difficult to be effective on brand-new individuals. To solve this problem, in recent years, Predictive Models have been proposed to build a generalized neuroimaging model, which is designed to predict individual observation and generalize to new individual data (Scheinost et al., 2019). Besides, convolutional neural network (CNN) has been widely used in neuroimaging, especially for the fMRI, for it can extract the local features which contribute to classification, prediction, and identification (Anwar et al., 2018). Therefore, in this work, we propose a novel hybrid learning framework that comprehensively studies both brain spatial (by Predictive Model) and temporal (by CNN model) characteristics *via* N-fMRI. Specifically, the whole brain functional spatial patterns under stimuli of three different audio categories (classic/pop/speech) are obtained *via* N-fMRI and serve as input to Predictive Model to achieve audio classification and significant spatial feature identification. Then, regions of significance (ROS) were generated based on the widely used AAL90 atlas (Rolls et al., 2020), and the significant spatial features identified by Predictive Model. Next, voxel-level signals in the ROS were extracted and fed to the one-dimensional CNN (1D-CNN) model to explore the voxels that consistently contribute to audio classification from the temporal perspective and investigate the characteristics of their temporal representations.

Our experimental results show that the proposed framework can achieve promising performance in naturalistic audio classification. Especially for distinguishing the classic and speech audios, the accuracy of classification is up to 92%. Based on the proposed framework, we can effectively characterize the spatiotemporal features of brain functional activity under N-fMRI. Brain regions in the temporary lobe and other regions related to audio are successfully identified, and signals of voxels in these regions are interpreted on spatiotemporal features. In addition, through qualitative temporal analysis of brain high-level semantic features and low-level acoustic features, we alleviate the semantic gaps from the view of frequency domain.

The key characteristics of this work can be summarized as three perspectives. First, an effective and hybrid learning computational framework, which integrates both brain spatial and temporal analysis, is proposed to study the brain states and high-level semantic features from N-fMRI. Second, ROS with spatial activation features across individuals and voxels of significance (VOS) with temporal characteristics were identified at a finer spatiotemporal scale. Third, “semantic gap” between high-level semantic features and low-level acoustic features is attempted to alleviate *via* our proposed framework.

The remainder of this article is organized as follows: Section “Materials and methods” briefly introduces the overall methods, including data acquisition, high-level feature extraction, Predictive Model, and 1D-CNN model. Section “Results” provides the experimental results, and section “Conclusion” concludes this article.

## Materials and methods

### Overview

**FIGURE 1:**
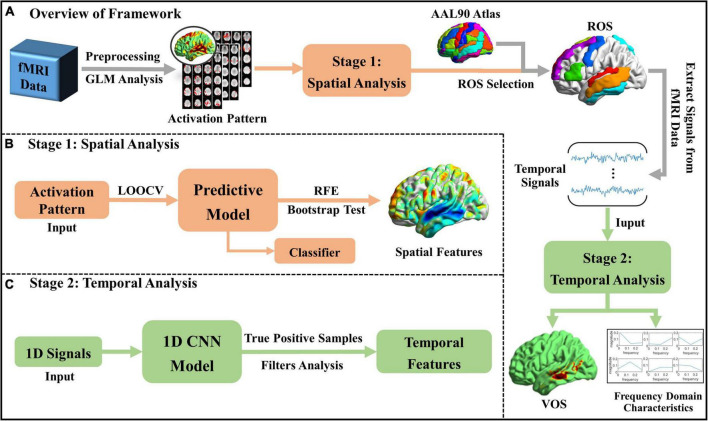
Workflow of the proposed framework. **(A)** The overview of the whole framework; **(B)** spatial analysis at Stage 1; and **(C)** temporal analysis at Stage 2.

Aiming at the fine-grained interpretation of spatiotemporal patterns of brain activities, workflow of the proposed framework is shown in Figure 1, which mainly includes two stages. At Stage 1, significant spatial features of brain activities corresponding to different naturalistic audio categories are identified *via* machine learning (ML) based Predictive Model, with the input of brain activation patterns extracted from N-fMRI data. Then, ROS are selected based on the AAL atlas (Rolls et al., 2020) and significant spatial features identified by Predictive Model, thus we focus on certain relevant regions instead of the whole brain. At Stage 2, voxel-level signals in the ROS are extracted and fed to 1D-CNN model to explore the voxels that consistently contribute to audio classification from the temporal perspective and investigate the characteristics of their temporal representations. Furthermore, the relationship between high-level semantic features and low-level acoustic features is explored.

### Data preprocessing and pattern extraction

#### Naturalistic audio data description

Three typical categories of music/speech: classical music (CLA), pop music (POP), and speech (SPE) are adopted as the stimulus materials in the dataset. For each category, 7 representative excerpts are selected for fMRI scanning. The excerpts are taken from legal copies of MP3 compressed audio files to ensure a variety of different recording qualities. Each excerpt is 90 s, totally making 3 *s*×7 s ×90 *s* = 31.5 min of audio data for each subject. Before the N-fMRI collection, we randomly compose the audio excerpts into two parts, each of which is about 15 min long. Between the two parts, participants could have a break with flexible duration.

#### Naturalistic functional magnetic resonance imaging data description

Collection of N-fMRI was conducted at the University of Georgia (UGA) under UGA Institutional Review Board (IRB) approval. Since fMRI scanning is costly and time-consuming, seven young healthy college students were recruited for this study and scanned in a GE 3T Sigma MRI system (GE Healthcare, Milwaukee, WI, USA) using an 8-channel head coil. The TR of this scan is set to be 1.5 s, and each excerpt collects 60 volumes of fMRI. Other parameters are as follows: 64 ×64 matrix size, 4 mm slice thickness, 220 mm Field of View (FOV), 30 axis slices, TE = 25 ms, and ASSET = 2. Note that for each subject and each excerpt, we obtained the 4D N-fMRI data with the size of (60, 91, 109, 91), where 60 is the number of volumes, and (91, 109, 91) is the size of one volume image.

#### Naturalistic functional magnetic resonance imaging data preprocessing

The preprocessing pipeline included motion correction, slice-timing correction, smoothing, registration, and normalization (Churchill et al., 2017) using FMRIB Software Library (FSL) (Jenkinson et al., 2012). To perform group-wise analysis on participants with a biological difference, all N-fMRI data are registered from individual space into the standard Montreal Neurological Institute (MNI) 152 standard space (Evans et al., 2012) by the FMRIB’s Linear Image Registration Tool (FLIRT) (Jenkinson and Smith, 2001).

#### Activation pattern extraction

To extract brain activation patterns, we adopted fMRI Expert Analysis Tool (FEAT) to conduct first-level General Linear Modeling (GLM) analysis by modeling task design corresponding to each 90 s naturalistic stimuli of each excerpt of an audio category (Woolrich et al., 2001). The preprocessed 4D N-fMRI data are adopted as input, and the 3D activation pattern with a size of (91, 109, 91) is obtained by GLM analysis. In total, there are 49 brain patterns (7 subjects × 7 excerpts) for one category of audios, which would serve as input to the Predictive Model.

### Spatial analysis with Predictive Model

To perform spatial analysis across individuals, Predictive Model, which is designed to predict individual observation and generalize to new individual data, is needed. Recently, Kohoutová et al. (2020) proposed an ML-based framework that consists of model-, feature-, and biology-level assessments to provide complementary results that support the interpretability of Predictive Model. Motivated by this framework, we adopt Predictive Model to analyze the N-fMRI data from a spatial perspective, striving to obtain the common significant spatial features across individuals. To focus on certain relevant regions from the whole brain, we select ROS based on AAL90 atlas (Rolls et al., 2020) and the significant spatial features identified by Predictive Model, so that we can better interpret those significant spatial features in the further analysis.

#### Procedure of Predictive Model

The major procedure of the Predictive Model for N-fMRI analysis includes extraction of brain activation patterns (input features of Predictive Model), selection of appropriate learning algorithms, training and classification, verification of classification performance, and identification of features related to classification. To establish an effective Predictive model for brain state differentiation during naturalistic stimuli, three key points are necessary to be determined: (1) Input Features; (2) Learning Algorithm; and (3) Training Strategy.

##### Input features

Activation patterns of the whole brain are adopted as the key features of input to the Predictive Model. In order to reduce the size of fMRI activation patterns, we adopted the commonly used MNI 152 T1 brain mask to extract the voxels within brain space in this work. MNI 152 T1 brain mask contains voxels with values of either “1” or “0.” Voxels with value “1” are located inside the brain, and voxels with value “0” are outside the brain. Since the fMRI data we adopted have been registered to the MNI 152 standard space, targeted voxels could be located and extracted by the mask. As a result, we reduced the voxel number of an activation pattern from about 910 thousand (91 × 109 × 91, whole space of fMRI image) to about 220 thousand (whole space of brain areas).

##### Learning algorithm

For the learning algorithms, multiple regression, LASSO regression, support vector machine (SVM), and support vector regression (SVR) are potential algorithms for classification and prediction (Ray, 2019). Among them, SVM is one of the most popular machine learning algorithms in current neuroimaging literature and has been proven to show promising performances on a small dataset (Scheinost et al., 2019). Considering the relatively small N-fMRI data in this work, SVM has a great advantage over other algorithms to obtain promising performance. Therefore, we adopted SVM to build the computing kernel in the Predictive Model. To fit the requirements of SVM, each sample with 220 thousand voxels was flattened into a one-dimensional vector. In this model, we selected the widely used linear kernel, which maps low-dimensional non-linear data to higher-dimensional space.

##### Training strategy

For the training strategy, we evaluated the performance of the Predictive Model on N-fMRI data by measuring the prediction accuracy. Here, we adopted Leave-One-Out-Cross-Validation (LOOCV), a commonly used method in machine learning, as the training strategy. For each binary classification task, the total number of available patterns is 98, corresponding to the 98 music excerpts (7 subjects × 7 excerpts × 2 categories). Depending on the LOOCV strategy, we treated each of the patterns as a sample, and samples of each participant, in turn, served as a testing set and samples of the rest participants as the training set. This process was totally repeated 7 (number of participants) times to reduce the size of the features with different combinations of training and testing data.

Moreover, to quantitatively illustrate the classification performance of the Predictive Model, we adopt the widely used statistical tool, receiver operating characteristic (ROC) curve (Obuchowski and Bullen, 2018), to describe the accuracy of the Predictive Model. As shown in Eq. 1, Sensitivity (*S*_*N*_) and Specificity (*S_P*) are a conditional probability of correctly identifying the true samples and false samples, respectively. The learned classifier with a low false positive rate and a high true positive rate suggests promising classification performance:


(1) $$\left\{ \begin{array}{c} S_{N}=\frac{T\,P}{T\,P+F\,N}\\ S_{P}=\frac{T\,N}{T\,N+F\,P} \end{array}\right.$$

where *TP*, *FN*, *FP*, and *TN* are the true positive samples, false negative samples, false positive samples, and true negative samples in predicted outcomes, respectively.

##### Comparison with baseline technologies

To adequately compare the classification performance, we set the comparison experiments from two perspectives: learning algorithms and feature extraction methods. For different learning algorithms, we chose methods of Support Vector Regression (SVR) and Principal Components Regression (PCR) as the baseline comparison. For different feature extraction methods, we chose the acoustic features (according to dynamics, rhythm, timber, pitch, and tonal) of the naturalistic audios and brain connectivity of N-fMRI as the baseline comparison, which was presented by Jiang et al. (2012).

#### Spatial features identification

To identify the significant spatial features from the classifier, we choose two commonly used methods: bootstrap tests (Erlikhman and Caplovitz, 2017) and recursive feature elimination (RFE) (Craddock et al., 2009), to identify features that are most related to the pattern classification. Bootstrap tests identify features that make stable contributions to prediction across participants. Features with stable weights in the classifier will be identified as significant features. RFE fits a model and removes the weakest features until the specified number of features is reached. During the iterative training procedure of Predictive Model, features corresponding to its lowest weights are eliminated from the training dataset until the optimal number of features is left. Theoretically, both bootstrap tests and RFE can identify significant spatial features in the Predictive Model.

#### Regions of significance selection

Considering that the significant spatial features identified by Predictive Model are distributed over multiple brain regions, in this section, we selected regions with the most significant spatial features as ROS for future temporal analysis based on AAL atlas (Rolls et al., 2020). Specifically, for each AAL region, we calculated the ratio of the voxel number of spatial features to the voxel number of all voxels, which are defined as *r_i* in Eq. 2, and then we selected ROS with a larger *r_i* from the whole brain:


(2) $$r_{i}=\frac{n}{m}$$

where *i* is the index of AAL brain region, *r* is the feature voxel proportion of a region, *n* is the voxel number of the significant spatial features in a region, and *m* is the total voxel number of a region.

### Temporal analysis with one-dimensional convolutional neural network

Although Predictive Model can conduct audio classification and provide related significant spatial features, the full representations of brain patterns are still to be explored. Based on the selected ROS, we further analyzed the temporal features of N-fMRI in this section. Considering the advantage of the CNN model to automatically extract the local features from the input samples, we further introduced 1D-CNN model for temporal analysis into the framework. In the 1D-CNN model, N-fMRI signals of voxels in ROS are extracted as the input with label of audio categorizes, the local temporal features that related to audio categorizes are effectively characterized.

#### One-dimensional convolutional neural network model architecture and experiment design

##### Architecture of one-dimensional convolutional neural network

**FIGURE 2:**
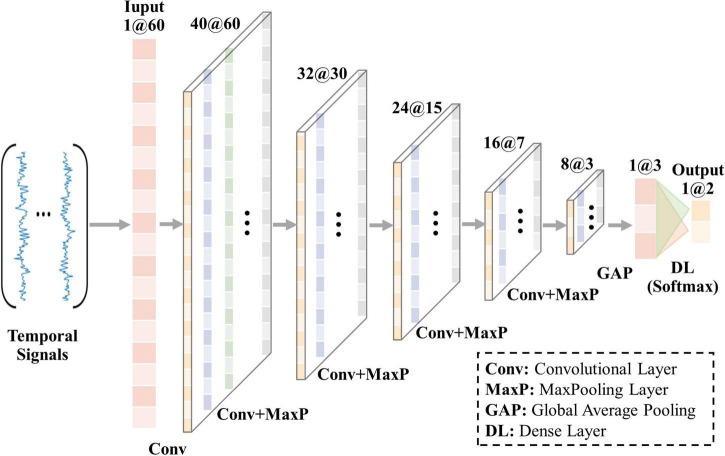
Architecture of 1D-CNN model in our framework.

In the previous research on resting state fMRI (rs-fMRI), four convolutional layers were adopted in 1D-CNN model and achieved promising classification performance (Zhang S. et al., 2019). Considering that signals in N-fMRI have richer features than those in rs-fMRI and single-task fMRI (Saarimäki, 2021), we set up five convolutional layers to train the model and extract hidden features from the input signals with a length of 60, as shown in Figure 2. Each of the last four convolutional layers is followed by a max-pooling layer, by which the output feature maps will be reduced to half the size of the input after passing through. At the end of 1D-CNN model is the dense layer (Softmax), whose input is the feature maps of input signals and output is the predictive result. In addition, architecture parameters are empirically set as follows: the number of the convolution filters is 40, 32, 24, 16, and 8 in each convolutional layer, respectively. The size of all kernels is 7, which is suitable for analyzing the features of signals with a length of 60. To keep the size of output feature maps the same as the input feature maps in each convolutional layer, we set the same padding and stride equal to *1*. Moreover, the optimizer is SGD, activation function is Relu, batchsize is 64, and learning rate is 1×10^−4^. The loss function is selected as “binary cross entropy.”

##### Experiment design

In this section, we conducted experiments using N-fMRI data from CLA and SPE tasks as examples and used 98 excerpts (7 subjects × 7 excerpts × 2 categories) of available N-fMRI data. We applied each ROS as a mask to extract voxel temporal signals from all the 98 excerpts of the preprocessed N-fMRI data and thus we possessed 98 excerpts of signals for each voxel in the studied ROS. Further, we adopted LOOCV training strategy in the 1D-CNN model. Specifically, for voxels of the studied ROS, we successively selected the signals of one excerpt as test samples and signals of other 97 excerpts as training samples. In this stage, we conducted the experiment on each of ROS separately, i.e., the number of separate parallel experiment is equal to the number of ROS. We finally obtain 98 sets of predicted results for voxels in each of the ROS.

#### Voxels of significance identification

In the 1D-CNN model, voxel signals with significant temporal characteristics could be classified with a higher classification accuracy, which can contribute to audio classification. Based on the LOOCV strategy described in section “One-dimensional convolutional neural network model architecture and experiment design,” there will be 98 runs for each voxel in each of the ROS. Here, we counted the true positive predicted results among the 98 sets of results for each voxel. If the ratio of number of true positive results to the 98 sets of results reaches a certain proportion (60% was chosen empirically) for a voxel, we consider that signals within this voxel are significantly related to the audio category and defined the voxel as VOS. Thus, VOS are identified in this stage. By studying the area that consists of VOS, we can explore how brain function works under naturalistic auditory stimuli at a finer spatial scale.

#### Exploration of relationship between high-level semantic features and low-level acoustic features *via* one-dimensional convolutional neural network model

“Semantic gap” between high-level semantic features (obtained from N-fMRI data) and low-level acoustic features (obtained from the audios) is a key problem in the N-fMRI study. Researchers have discovered more and more frequency-specific biological interpretations from fMRI (Yuen et al., 2019). In this section, the proposed framework may bring new insights into alleviating the “semantic gap” by analyzing frequency domain features of N-fMRI and audios.

In the 1D-CNN model, the convolution kernels are constantly trained and optimized in the process of convolution with input signals. The significant features of voxel signals that contribute to the classification of audio categories are embedded in those kernels (Huang et al., 2018). In this article, we selected the convolution kernels in the last convolutional layer to explore which category they are related to. To be specific, feature maps generated by convolution kernels in the last convolutional layer connect to the dense layer, thus each of these convolution kernels can be mapped to a pair of weights, which are related to the prediction result of audio categories (Lin et al., 2013). Therefore, we extracted the values of convolution kernels in the last convolutional layer and the weights in the dense layer, and then established a one-to-one relationship between kernels and categories (Liu et al., 2019; Zhang S. et al., 2019). By the analysis of convolution kernels, we can generate the temporal features of N-fMRI related to different audio categories in each region separately.

**FIGURE 3:**
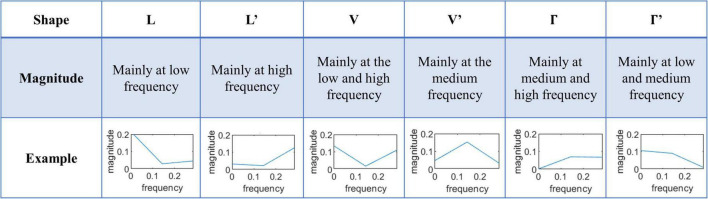
The six typical shapes of frequency domain features of convolution kernels.


To identify and interpret the difference in temporal features of fMRI, the learned convolution kernels could be transferred into the frequency domain to explore the frequency characteristics of fMRI (Liu et al., 2019; Zhang S. et al., 2019; Jiang et al., 2020). Since the size of the convolution kernels at the temporal domain is 7, the frequency domain includes three points, i.e., about half size of the kernel, as shown in Figure 3. To efficiently and comprehensively study the shape of the features from the frequency domain, six typical types are observed and defined as *L*, *L*′, *V*, *V*′, Γ, and Γ′, which are shown in Figure 3. Moreover, the six shapes are mathematically defined in Eq. 3:

(3) $$\left\{ \begin{array}{c} L\;:\;X\;\left(1\right)>m\,e\,a\,n\;\left(X\right)\;,\;X\;\left(2\right)<m\,e\,a\,n\,\;(X)\;,\;X\;\left(3\right)<m\,e\,a\,n\,\;(X)\\ L^{\prime }\;:\;X\;\left(1\right)>m\,e\,a\,n\,\;(X)\;,\;X\;\left(2\right)<m\,e\,a\,n\,\;(X)\;,\;X\;\left(3\right)<m\,e\,a\,n\,\;(X)\\ V\;:\;X\;\left(1\right)>m\,e\,a\,n\,\;(X)\;,\;X\;\left(2\right)<m\,e\,a\,n\,\;(X)\;,\;X\;\left(3\right)<m\,e\,a\,n\,\;(X)\\ V^{\prime }\;:\;X\;\left(1\right)>m\,e\,a\,n\,\;(X)\;,\;X\;\left(2\right)<m\,e\,a\,n\,\;(X)\;,\;X\;\left(3\right)<m\,e\,a\,n\,\;(X)\\ \Gamma \;:\;X\;\left(1\right)>m\,e\,a\,n\,\;(X)\;,\;X\;\left(2\right)<m\,e\,a\,n\,\;(X)\;,\;X\;\left(3\right)<m\,e\,a\,n\,\;(X)\\ \Gamma^{\prime }\;:\;X\;\left(1\right)>m\,e\,a\,n\,\;(X)\;,\;X\;\left(2\right)<m\,e\,a\,n\,\;(X)\;,\;X\;\left(3\right)<m\,e\,a\,n\,\;(X) \end{array}\right.$$

where *X*(*n*) represents the *n*th value in the *X*-shape frequency domain array.

For the low-level acoustic features, we calculated two typical acoustic features: mel frequency cepstral coefficients (MFCCs) (Grama and Rusu, 2017) and Spectral Centroid (Prasetio et al., 2021).

Mel frequency cepstral coefficients are coefficients that collectively make up a Mel-Frequency Cepstrum (MFC), which have been widely used in automatic speech and speaker recognition. Specifically, the human ears in listening act like filters, which are better at identifying small changes in audio at lower frequencies (blow 1000 Hz) but not good at higher frequencies (higher than 1000 Hz). Mel-scale is a scale that relates the human perceived frequency to the actually measured frequency *f*. The formula to convert frequency *f* to Mel-scale *Mel*(*f*) is illustrated in Eq. 4 (Gupta et al., 2013). MFCCs are commonly derived as follows: (1) Take the Fourier transform of the audio signal; (2) Map the powers of the spectrum obtained above onto the Mel-scale; (3) Take the logs of the powers at each of the Mel frequencies; (4) Take the discrete cosine transform of the list of Mel log powers; and (5) The MFCCs are the amplitudes of the resulting spectrum:


(4) $$M\,e\,l\,\left(f\right)\;=\;2595\times l\,g\,\left(1\;+\frac{f}{700}\right)$$

where *f* is the actual measured frequency, *Mel*(*f*) is the Mel-scale, 2,595 and 700 are the commonly used constants in Mel-scale formula.

Spectral Centroid is one of the important physical parameters describing the properties of timber, which indicates where the centroid of the spectrum is located (Prasetio et al., 2021). Generally, the audios with dark and deep quality tend to have more low-frequency components and relatively low Spectral Centroid, while the audios with bright and cheerful quality mostly concentrate on high frequency and relatively high Spectral Centroid. It is calculated from the Fourier transform frequency and amplitude information, as defined in Eq. 5:


(5) $$C\,e\,n\,t\,r\,o\,i\,d=\frac{\sum_{n\;=\;0}^{N-1}f\;\left(n\right)\;X\;\left(n\right)}{\sum_{n\;=\;0}^{N-1}\;X\;\left(n\right)}$$

Where *x*(*n*) represents the weighted frequency value or magnitude of bin *n*, and *f*(*n*) represents the center frequency of *n*.

## Results

In our experiment, the Predictive Model is configured based on CanlabCore toolbox, which is available at https://github.com/canlab/CanlabCore. The 1D-CNN model is built based on Keras (a deep learning application programming interface), which runs on top of the machine learning platform TensorFlow 2.6.0 (Géron, 2019). The computing environment is a server with NVIDIA Geforce GTX 3090 with 24 GB GPU.

### Characterizing spatial patterns *via* Predictive Model

#### Visualization of input features for Predictive Model

**FIGURE 4:**
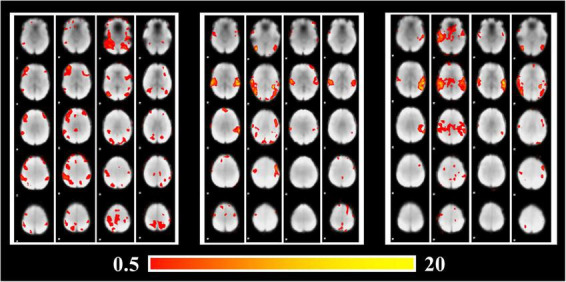
Brain activation patterns under stimuli of different audio categories.

As the key features fed into the Predictive Model, activation patterns obtained by GLM analysis are visualized in Figure 4. As shown in Figure 4, the activation regions of POP and SPE are relatively similar, especially in the Temporal lobe which contains significant activations. However, the activation patterns of CLA are significantly different from that of POP and SPE, which include parts of the Frontal lobe and Parietal lobe. In terms of audio components, CLA audios only have instrumental sounds without vocals, whereas POP and SPE both have vocals. It may reveal the attention mechanism of Temporal lobe, which focuses more on the vocals than other sounds.

#### Classification performance of Predictive Model

**FIGURE 5:**
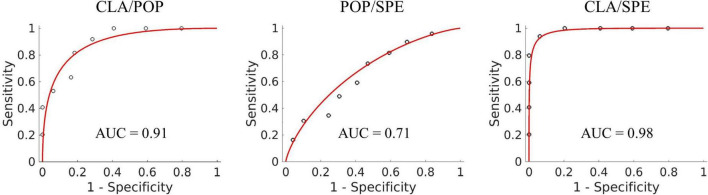
ROC curve of three binary classification tasks.

We visualized the ROC curves for the classification results of the Predictive Model for classification performance assessment. Figure 5 shows the ROC curve of three binary classification tasks, in which the accuracy of CLA/POP, POP/SPE, and CLA/SPE is 81, 62, and 92%, respectively. The results support our justification in section “Visualization of input features for Predictive Model” that activation patterns of POP and SPE are relatively tough to be distinguished and activation patterns of CLA are easier to be identified. Besides, the sensitivity and specificity are 80 and 82% for CLA/POP task, 73 and 51% for POP/SPE task, and 96 and 88% for CLA/SPE task, respectively. In POP/SPE task, the specificity of 51% indicates that the probability of misjudging negative samples is high, which is the main reason for low classification accuracy. Moreover, in order to further explore the difference among three audio categories, we provide the results of three classifications, for details please refer to the Supplementary material.

Besides, the results of the baseline comparison are provided in Table 1. When comparing with different learning algorithms, SVM performs best in CLA/POP and POP/SPE classification task and generates well performance in CLA/SPE classification task. When comparing with different feature extraction methods, features of whole brain activation perform better than both functional connectivity and acoustic features. Overall, SVM performs the most robustness classification performance and features of whole brain activation generate the best classification performance among these feature extraction methods.

**TABLE 1:** Comparison of different learning algorithms and feature extraction methods.

Comparison	Algorithm/method	Accuracy		
		CLA/POP	POP/SPE	CLA/SPE
This article	Whole brain activation/SVM	**83** ±**3.8%**	**65** ±**4.8%**	91 ±2.9%
Different learning algorithms	Whole brain activation/SVR	73 ±4.5%	56 ±5.0%	**96** ±**2.0%**
	Whole brain activation/PCR	71 ±4.6%	55 ±5.0%	94 ±2.4%
Different feature extraction methods	Functional connectivity matrix/SVM	70.5%	63.5%	75%
Jiang et al., 2012	Acoustic features/SVM	52.5%	49.5%	63.5%

#### Exploration of the significant spatial features

**FIGURE 6:**
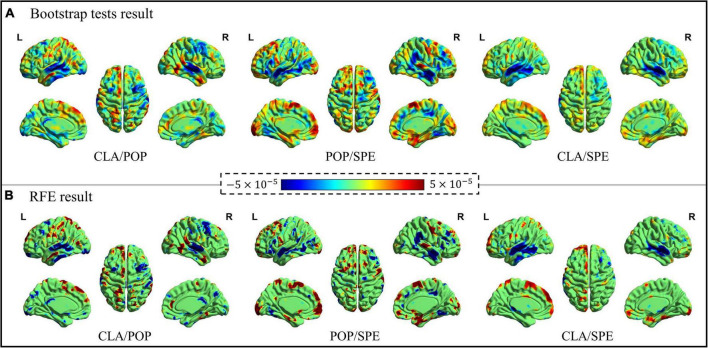
Bootstrap tests and RFE results of significant spatial features. **(A)** Significant features learned by Bootstrap tests, and **(B)** significant features learned by RFE.

Significant spatial features identified from the bootstrap tests and RFE procedure were visualized in Figure 6 *via* the BrainNet Viewer (Xia et al., 2013). As shown in Figure 6, the positive activation (color “red”) represents the meaningful features of the former category, the negative activation (color “blue”) represents the meaningful features of the latter category. It is worth noting that both the positive and negative features contribute to the classification.

Figure 6 demonstrates that the spatial maps of bootstrap tests and RFE results are similar, consistently demonstrating the effectiveness of the Predictive Model for spatial feature identification. Although the results of bootstrap tests cover more brain areas, the activation features from RFE provide a stronger contrast between two categories with less area. Moreover, for the classification of CLA/POP and CLA/SPE, activation features are more concentrated on specific locations, while positive and negative activation areas stay away from each other. However, it is quite clear that positive and negative activation areas overlap for the classification of POP/SPE, indicating that even at the semantic level, POP and SPE are still hard to be distinguished.

#### Identification of regions of significance

**FIGURE 7:**
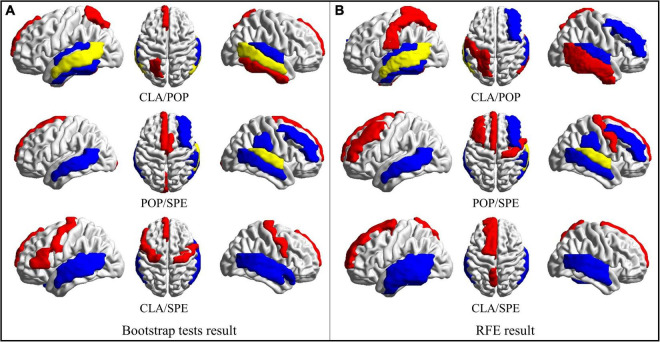
Regions of brain where activation is significant. **(A)** ROS selected by Bootstrap test results; **(B)** ROS selected by RFE results. (Red represents areas with positive activation, blue represents areas with negative activation, and yellow represents areas with both positive and negative activation).

Based on the obtained bootstrap tests and RFE results, brain regions with the most positive and negative activation features are counted and then visualized in Figure 7 *via* the BrainNet Viewer (Xia et al., 2013). From Figure 7, we found that some brain areas are related to certain classical categories, like “superior frontal gyrus medial,” as shown in red circle. We also found that some areas are related to more than one category. For example, both pop and speech categories are related to the “superior temporal gyrus,” which is the yellow region shown in POP/SPE result in Figure 7. Both classical and speech categories cause “middle temporal gyrus” to activate, which is the yellow region shown in CLA/POP result in Figure 7. These results reveal an interesting phenomenon that some certain brain regions are consistently related to the specific category of audios, while some are merely activated by a certain category of audios.

**FIGURE 8:**
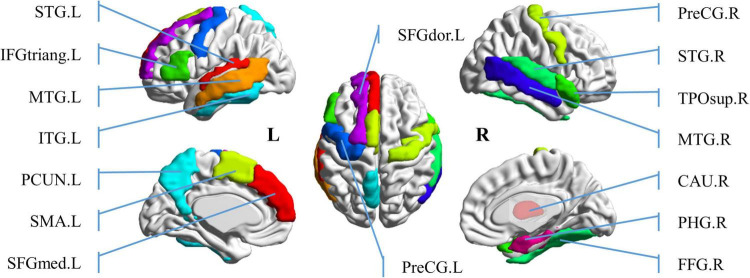
Visualization of top 16 brain regions containing the most significant spatial features.

In order to further study the spatial and temporal characteristics of ROS, CLA/SPE categories, which provide the best classification performance in Predictive Model, are selected as the test bed for further analysis. For each audio category and each significant feature identification method, we selected the top 6 regions with the most significant features (total 24 regions). Table 2 shows the regions with the most significant features selected by Bootstrap tests and RFE in Predictive Model. From Table 2 we can see that regions selected by Bootstrap tests and RFE both have difference and intersection. To identify significant spatial features as much as possible and further select comprehensive brain regions for the temporal analysis in the following section, we combined the regions (total 16 regions) learned by the two methods and visualized the 16 ROS associated with CLA/SPE, as shown in Figure 8. To simplify the expression, we refer to each region in the format of the abbreviations in the following sections. For the full name and abbreviations of regions involved in this article, please refer to the Supplementary material.

**TABLE 2:** Regions with most spatial features identified by Bootstrap tests and RFE.

Audio category	Positive (CLA)			Negative (SPE)		
Methods	Bootstrap		RFE	Bootstrap		RFE
Division	SFGmed.L IFGtriang.L PreCG.L PHG.R PreCG.R FFG.R		SFGmed.L SMA.L PCUN.L SFGdor.L PHG.R FFG.R	MTG.L STG.R MTG.R STG.L TPOsup.R ITG.L		MTG.L STG.R MTG.R STG.L ITG.L CAU.R
Intersection	SFGmed.L PHG.R FFG.R			MTG.L STG.R MTG.R STG.L ITG.L		

### Characterizing spatiotemporal patterns *via* one-dimensional convolutional neural network

#### Effectiveness of one-dimensional convolutional neural network model *via* model evaluation experiment

**FIGURE 9:**
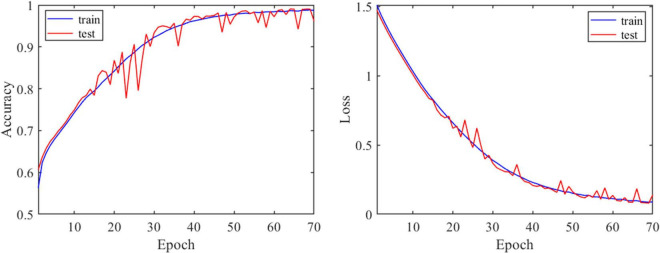
Performance of 1D-CNN model *via* model evaluation experiment.

To verify the validity of 1D-CNN model, MTG. L brain region was randomly chosen to perform the validation experiment. N-FMRI signals from MTG. L brain region of all participants during CLA and SPE tasks were extracted as the dataset, which was then divided into the training set and testing set followed by the ratio of 4:1. Both the training and testing classification performances are shown in Figure 9. It can be seen that with the increase in epoch, the accuracy of training and testing both increased up to about 97%, and the corresponding loss value decreased and converged from 1.5 to less than 0.1. The performance indicates that the proposed 1D-CNN model has satisfying performance in classifying CLA and SPE signals.

#### Interpreting the voxels of significance

**FIGURE 10:**
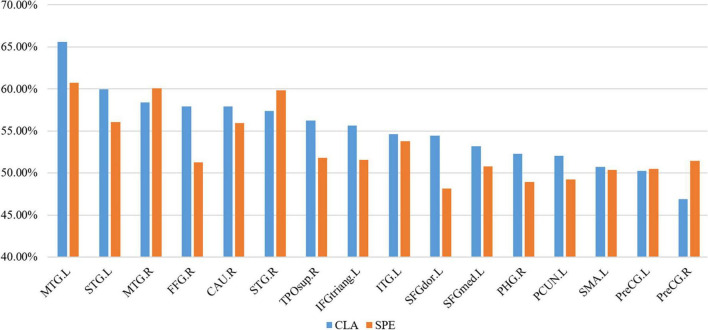
The proportion of VOS in each ROS.

For the 16 selected ROS, we calculated the proportion of VOS number to the total voxel number in both CLA and SPE tasks. As shown in Figure 10, the proportion of VOS is more than 50% in almost all brain regions. Especially, the proportions of VOS exceed 55% on MTG. L, MTG. R, STG. L, STG. R, and CAU. R in both CLA and SPE audio tasks. The brain regions with a higher proportion of VOS, which mostly spread over the temporal lobe, such as MTG.L, MTG.R, STG. L, and STG. R, are consistent with common perception and research (Whitehead and Armony, 2018). Besides, the proportions of VOS on MTG.L, STG.L, FFG.R, TPOsup. R, IFGtriang. L, and SFGdor. L in CLA task are 4% greater than that in SPE task, indicating that CLA signals have more unique and significant characteristics than SPE signals in these regions. In addition, the proportions of VOS brain regions outside the temporal lobe, such as FFG. R and CAU. R, are more than 55% in CLA task, which may help reveal the non-auditory function of these brain regions.

**FIGURE 11:**
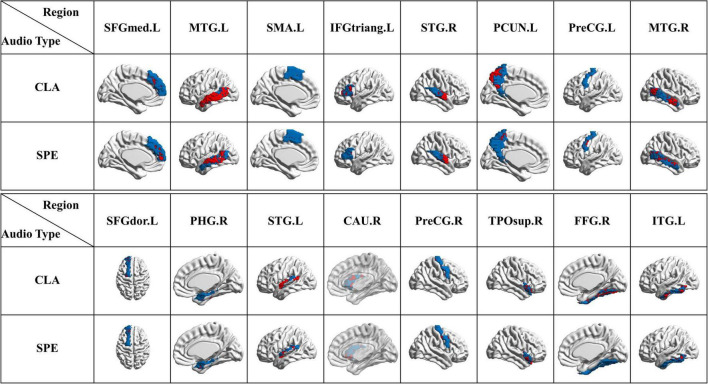
Visualization of VOS in each ROS (blue represents each single brain region, and red represents the collection of VOS in each region).

To achieve a more intuitive understanding and interpreting of the significant voxels, we selected and visualized the VOS in each brain area in Figure 11, where the blue represents each region, and the red represents the collection of VOS in each region. We can see that most of the red areas spread over the temporal lobe, such as MTG. L, MTG. R, STG. L, and STG. R. We can also see that red area of MTG. L is about 5% larger than that of MTG. R for CLA signals, indicating the activation characteristics of MTG. L region (on left brain) were more consistent and dominant than MTG. R region (on right brain), as shown in Figures 10, 11. This further supports the phenomenon that the auditory function of the left brain is greater than that of the right brain. Besides, FFG. R also has distinct activation characteristics. Although existing studies could not conclusively prove the exact auditory-related function of FFG. R, its performance obtained by our framework may help reveal it.

#### Relationship between high-level semantic features and low-level acoustic features

In order to analyze the high-level semantic features in N-fMRI, we calculated the frequency domain features of voxel signals. As introduced in section “Exploration of relationship between high-level semantic features and low-level acoustic features *via* one-dimensional convolutional neural network model,” the convolution kernels containing key features are divided into six types according to the frequency domain distribution. Since kernels of V and V’ shape contain both low- and high-frequency domains, the analysis of V and V’ shape would be more complex than that of other four types with a single frequency state (higher or lower). Therefore, we only focus on the four shapes except for V and V’ shape in this section.

**FIGURE 12:**
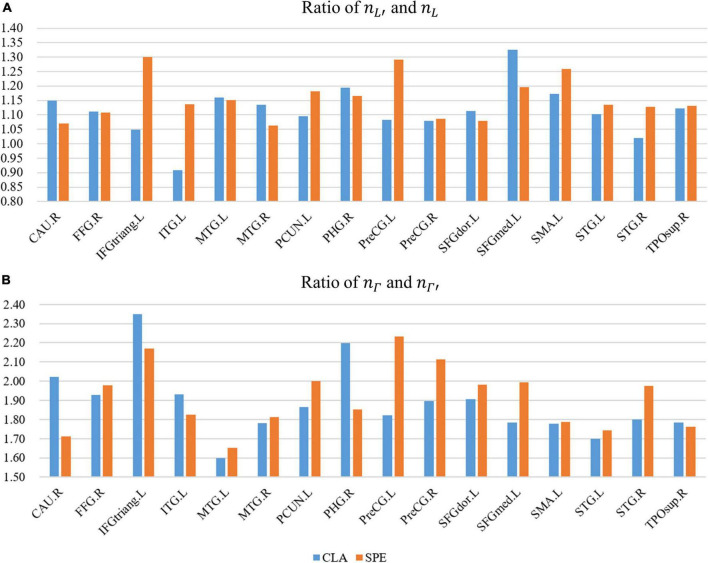
The ratio of higher frequency kernels to lower frequency kernels. **(A)** The ratio of the ratio of *n*_*L’*_ (higher) to*n_L_* (lower). **(B)** The ratio of *n*_Γ_ (higher) to$n_{\Gamma^{\prime }}$ (lower).

Firstly, the number of convolution kernels with higher and lower frequency domains was counted, respectively, represented as *n*_Γ_ (higher),$n_{L^{\prime }}$ (higher),*n_L_* (lower), and$n_{\Gamma^{\prime }}$ (lower). Then, we calculated the ratio of *n*_Γ_ (higher) to$n_{\Gamma^{\prime }}$ (lower), the ratio of *n*_*L’*_ (higher) to*n_L_* (lower), which represents the ratio of higher frequency kernels to lower frequency kernels, as shown in Figure 12. From Figure 12A, we find that the ratios in all regions except ITG. L are greater than 1, indicating the number of high-frequency convolution kernels is more than that of low-frequency convolution kernels in almost all ROS. From Figure 12B, we can see that ratios in all regions are greater than 1.6, even than 2 in the region of IFGtriang. L in both CLA and SPE. These results indicate that the high-frequency features of voxel signals are richer than the low-frequency features.

Furthermore, the ratio of *n*_*L’*_ (higher) to*n_L_* (lower) in SPE is 4.1% higher than that in CLA in Figure 12A. The ratio of *n*_Γ_ (higher) to$n_{\Gamma^{\prime }}$ (lower) in SPE is 2.8% higher than that in CLA in Figure 12B, indicating that signals of ROS in SPE have more high-frequency characteristics than that in CLA in total. Besides, in regions of PCUN. L, PreCG. L, PreCG. R, STG. L, and STG. R, the ratios in Figures 12A,B of SPE are both higher than that of CLA, where the values of difference are between 0.04 and 0.4. This result further discloses that voxel signals of SPE have consistently richer high-frequency characteristics than that of CLA in these regions.

**FIGURE 13:**
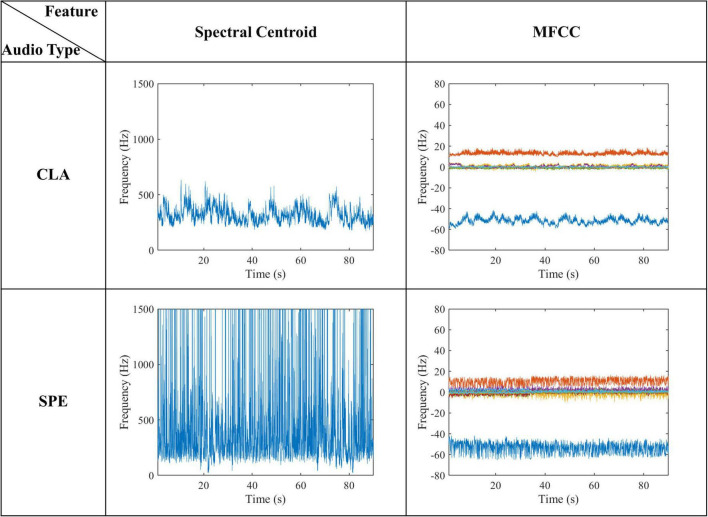
Low-level acoustic features of naturalistic audio.

Figure 13 shows two typical acoustic features extracted from naturalistic audios. As can be seen from Figure 13, the average Spectral Centroid of the SPE and CLA audios is about 900–1100 Hz and 400–500 Hz, respectively. For the MFCCs features, frequency of CLA audios is concentrated in several frequency bands with a range of no more than 10 Hz. Although the frequency of SPE audios is also concentrated in several frequency bands, the band oscillates a lot with a maximum range over 20 Hz. These two typical acoustic features indicate that the frequency domain of the SPE is not only higher than that of the CLA but also more volatile. By comparing low-level acoustic features with high-level semantic features, we find that the difference between SPE and CLA audio is consistent. We hypothesize that the audio with the larger Spectral Centroid leads to more intense brain activity. In terms of MFCCs features, the change in a frequency band may lead to more high-frequency characteristics in voxel signals.

## Conclusion

In this work, we propose a novel hybrid learning framework that comprehensively studies the brain spatial (*via* Predictive Model) and temporal (*via* CNN model) characteristics during N-fMRI. By integrating spatial and temporal characteristics, ROS are obtained *via* the Predictive Model, and VOS are further interpreted *via* 1D-CNN model. Experiment results show that the proposed framework can achieve promising classification performance of audio categories and identify meaningful characteristics of the high-level semantic features. Especially for the classic and speech audios, the accuracy of classification is up to 92%. Furthermore, the relationship between high-level semantic features and low-level acoustic features is proved to be consistent in the frequency domain. In conclusion, the proposed framework provides novel insights on characterizing spatiotemporal patterns from the N-fMRI and effectively studying the high-level semantic features under naturalistic stimuli, which will further benefit the understanding of the brain working mechanism and the advance of naturalistic stimuli clinical application.

## Data availability statement

The original contributions presented in the study are included in the article/Supplementary material, further inquiries can be directed to the corresponding author.

## Ethics statement

The studies involving human participants were reviewed and approved by the Institutional Review Board (IRB), University of Georgia (UGA). The patients/participants provided their written informed consent to participate in this study.

## Author contributions

SY, SZhang, and XJ: conception and design. SY: analysis and interpretation. TL: data collection. SY, SZhang, and XJ writing the manuscript. SY, SZhang, ES, RW, SZhao and XJ: critical revision of the manuscript. SZhang: overall responsibility. All authors contributed to the article and approved the submitted version.
