# Supplementary material for: A hybrid learning framework for fine-grained interpretation of brain spatiotemporal patterns during naturalistic functional magnetic resonance imaging
Source: Front Hum Neurosci. 2022 Sep 30;16:944543. doi: 10.3389/fnhum.2022.944543 (PMC9563232; doi:10.3389/fnhum.2022.944543)
Supplement: Supplementary file 1 [file Presentation_1.PDF]

Supplemental Materials

1. Experiment Results

In our work, three typical categories of music/speech: classical music (CLA), pop music (POP), and speech (SPE), are adopted as the stimulus materials in the dataset. To further explore the difference among three audio categories, we provide the results of three classification based on Predictive Model. The result of three classification is shown in **Table I**, with the corresponding confusion matrix shown in **Fig. 1**. From **Table I** we could see that, among the three categories of audios, performance of classifying CLA performs best, followed by POP and SPE, suggesting that CLA could be easily identified from POP and SPE. Refer to the confusion matrix of three classification results, it is clear that up to 57.14% of actual SPE audios are wrongly predicted as POP. However, for the actual POP audios, the percentages of wrongly predicted as CLA and SPE audios are 14.24% and 14.29% respectively, which are nearly the same. This indicates that N-fMRI data of POP may contain typical features of SPE, but the latter doesn't contain the typical features of the former.

Table I. Accuracy of three classification

Category	CLA	POP	SPE
Accuracy	87.76%	73.47%	40.82%

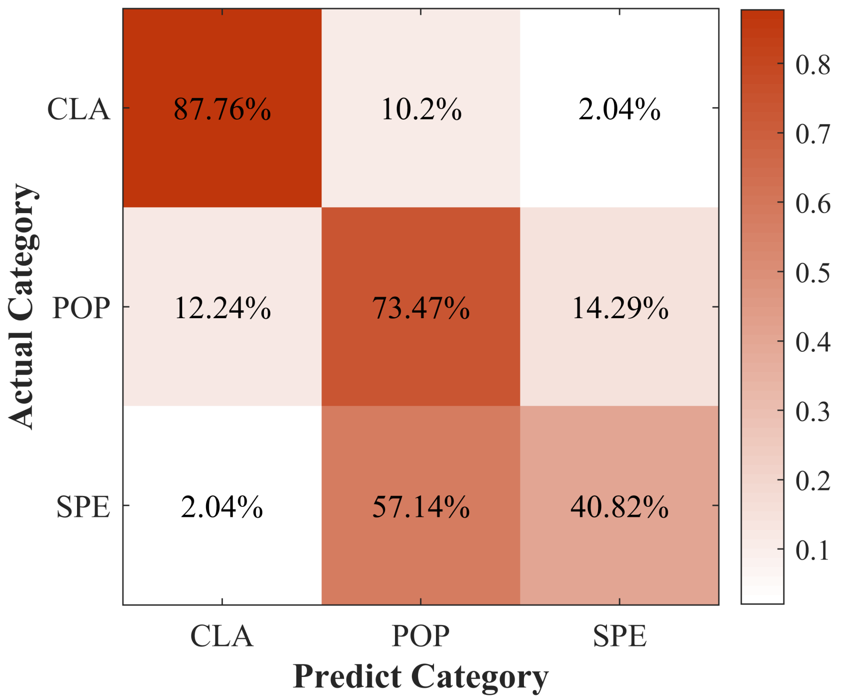

Figure 1. Confusion matrix of three classification results

2. Appendix

To simplify the expression, we refer to each region in the format of the abbreviations in the manuscript. For the full name and abbreviations of regions involved in this paper, please refer to **Table II.**

Table II. Full name and abbreviations of regions involved in this paper

NO.	Full name	Abbreviations	NO.	Full name	Abbreviations
1	Superior frontal gyrus, medial	SFGmed.L	9	Superior frontal gyrus, dorsolateral	SFGdor.L
2	Middle temporal gyrus	MTG.L	10	Parahippocampal gyrus	PHG.R
3	Supplementary motor area	SMA.L	11	Superior temporal gyrus	STG.L
4	Inferior frontal gyrus, triangular part	IFGtriang.L	12	Caudate nucleus	CAU.R
5	Superior temporal gyrus	STG.R	13	Precental gyrus	PreCG.R
6	Precuneus	PCUN.L	14	Temporal pole: superior temporal gyrus	TPOsup.R
7	Precental gyrus	PreCG.L	15	Fusiform gyrus	FFG.R
8	Middle temporal gyrus	MTG.R	16	Inferior temporal gyrus	ITG.L
